# Inducing Mild Traumatic Brain Injury in *C*. *elegans* via Cavitation-Free Surface Acoustic Wave-Driven Ultrasonic Irradiation

**DOI:** 10.1038/s41598-019-47295-1

**Published:** 2019-09-04

**Authors:** Morteza Miansari, Meghna D. Mehta, Jan M. Schilling, Yuta Kurashina, Hemal H. Patel, James Friend

**Affiliations:** 1 Medically Advanced Devices Laboratory, Center for Medical Devices, Department of Mechanical and Aerospace Engineering, University of California San Diego, 9500 Gilman Drive MC0411, La Jolla, 92093 CA United States of America; 2 Department of Mechanical Engineering, Babol Noshirvani University of Technology, P.O. Box 484 Babol, Iran; 3 Department of Cancer Medicine, Cell Science Research Center, Royan Institute for Stem Cell Biology and Technology, ACECR, Babol, Iran; 4 Department of Anesthesiology, University of California San Diego, 9500 Gilman Dr MC0801, La Jolla, 92093 California USA; 5 Present Address: School of Materials and Chemical Technology, Tokyo Institute of Technology, Nagatsuta, Yokohama 4259 Japan

**Keywords:** Neurological models, Stress and resilience, Biomedical engineering

## Abstract

Mild traumatic brain injury is an all-too-common outcome from modern warfare and sport, and lacks a reproducible model for assessment of potential treatments and protection against it. Here we consider the use of surface acoustic wave (SAW) irradiation of *C*. *elegans* worms—without cavitation—as a potential, ethically reasonable animal-on-a-chip model for inducing traumatic brain injury in an animal, producing significant effects on memory and learning that could prove useful in a model that progress from youth to old age in but a few weeks. We show a significant effect by SAW on the ability of worms to learn post-exposure through associative learning chemotaxis. At higher SAW intensity, we find immediate, thorough, but temporary paralysis of the worms. We further explore the importance of homogeneous exposure of the worms to the SAW-driven ultrasound, an aspect poorly controlled in past efforts, if at all, and demonstrate the absence of cavitation through a change in fluids from a standard media for the worms to the exceedingly viscous polyvinyl alcohol. Likewise, we demonstrate that acoustic streaming, when present, is not directly responsible for paralysis nor learning disabilities induced in the worm, but is beneficial at low amplitudes to ensuring homogeneous ultrasound exposure.

## Introduction

The increased incidence of reported blast-related head injuries has made traumatic brain injury (TBI) the signature injury of modern warfare^1^. TBI does not discriminate by age, socioeconomic group, nor gender, and as a significant health and economic burden^2,3^, it affects over 1.7 million people in the United States each year^4^. TBI can produce a wide array of acute symptoms in moderate-to-severe exposure, but blast-induced *mild* traumatic brain injury (bi-mTBI) is characterized by the distinct absence of acute clinical abnormalities. Although exact figures are unknown, approximately one in five wounded soldiers suffers from TBI^5^, and an estimated 52% of those injuries are bi-mTBI that occur over both short- and long-term scales^6–8^. In both civilian and military environments, exposure to a blast may cause instant death, injuries with immediate sign of symptoms, or concealed injuries manifesting over years to decades after the initial blast exposure^9^. Bi-mTBI may underlie a risk of later developing neurodegenerative disorders, such as Alzheimer’s disease^10,11^, chronic traumatic encephalopathy^12,13^, depression^14–20^, and neural, axonal, and glial tissue injury^21,22^.

The injuries suffered as a result of the blast exposure may serve to define the mechanisms of brain injury. Bi-mTBI is divided into four subcategories: the primary injury is the result of the direct effect of a blast wave, the secondary injury is due to the victim’s physical contact with blast-scattered fragments, the tertiary injury results from acceleration of the head due to wind from the blast and being struck by objects, and chemical and thermal blast effects deliver the quaternary injury^23–26^.

Unfortunately, bi-mTBI—the least understood damage mechanism caused by exposure to the primary blast wave^27^—may not show diagnostic signs of injury while still leading to serious long-term consequences^11,28–32^. Early detection and intervention could potentially alleviate or prevent later development of significant neurological dysfunction, especially in military veterans who do not actually present with signs of traumatic brain injury^33^. Therefore, there is an urgent need to better understand the underlying mechanisms of this injury due to the complexity of both the injurious environment and the resulting injuries. To this end, development of low-cost, high-throughput TBI models absent today would be beneficial in testing potential diagnostic and treatment technologies and help discover and palliate bi-mTBI.

As proposed by Cernak and Noble-Haeusslein^34^, an experimental blast-related TBI model may properly simulate the clinically relevant symptoms observed in humans if it satisfies the following criteria: the instrument of injury and the injury itself should mimic blast-induced neurotrauma and be quantifiable, controllable, and reproducible. The outcomes of the injury in terms of behavioral, biochemical, or physiological changes should correlate with the mechanisms of injury.

To this end, it is essential to have a biological model that mimics the human condition for studying the biomechanics, cellular and molecular aspects of human bi-mTBI, and preclinical development of TBI therapies. Furthermore, bi-mTBI has been shown to cause clinical symptoms such as short-term memory loss and learning problems in animals^12,33^.

A variety of TBI models in animals have been devised over the past decade to represent clinically relevant brain injuries in humans^35–41^. Fluid percussion, controlled cortical impact, direct impact acceleration, and blast exposure are perhaps the most common in the literature^24^. These methods are universally applied to mammals—from mice to cats, dogs, and monkeys—while having their own advantages and disadvantages. Fluid exposure requires a craniotomy but produces reproducible results. Direct impact with weights also typically requires a craniotomy but exhibits irreproducibility and substantial mortality; controlled cortical impact still requires the craniotomy but substantially improves the reproducibility. Of all these choices, a blast injury would perhaps seem the most obvious choice given its similarity to humans’ experience on the battlefield, yet it is not generally reproducible nor standardized. This may be the reason that a significant number of clinical drug trials fail after seeing potential preclinical benefit with blast injury exposure testing^42^.

All of these models require significant preparation time and the requisite ethics approvals, and are expensive to employ given the large number of animals required to discern biologically relevant effects, the protracted effort necessary to perform behavioral tests, and generally low throughput. These problems collectively limit progress in the pursuit of better treatment. In early research on therapeutic methods and drugs, it is important to fail quickly and find promising routes to possible success among the many dead ends. Non-mammalian organisms are often used at this stage. Of the animal models, *Caenorhabditis elegans* (*C*. *elegans*) is certainly a rapid, ethical, and inexpensive model that may be adapted to suit medium to high-throughput technologies^43–45^. We use *C*. *elegans* in this study as an alternative model organism to vertebrate animals (e.g., mice, rats, pigs).

For many reasons, *C*. *elegans* is a powerful model for biological research, ever since Brenner’s original proposition in 1963 to use *C*. *elegans* in neuronal development studies^46^. *C*. *elegans* offers a surprisingly large number of useful similarities to humans in cellular and intracellular function^47^. With 118 neuronal subtypes^48^ in 302 neurons representing the *connectome*^49^, *C*. *elegans* is a useful neuronal injury *in vivo* model thoroughly resolved to single neurons. Furthermore, *C*. *elegans* is ethically easy to use in high-throughput experiments and inexpensive to grow and in large numbers in the laboratory. A generation of *C*. *elegans* may be produced and advance from youth to adulthood in three days to bear a new generation of 300 or more animals from a single hermaphroditic parent^50^, who will progress onward to old age and death, all within twelve to eighteen days^51^. *C*. *elegans* is an especially convenient model in rapid trials requiring study of conditions that may arise only after significant aging, such as some of the effects of bi-mTBI. From an injury in youth, one may assess the long-term effects to old age in *C*. *elegans* in a single week. Its transparency facilitates straightforward observation of its cells in all growth stages, the many knockout and overexpressed genetic mutants, complete cell lineage, and established genetic characterization techniques in s all provide a variety of options to manipulate and study *C*. *elegans* at the molecular level. Consequently, the comprehensive study of TBI and potential treatments to reduce its impact in humans would be far faster and straightforward in this worm model than with current TBI animal models.

Recently, several reports describe the effects of ultrasound and shock waves on the behavior of *C*. *elegans*^52–54^. However, so far it appears that exposure of worms to artificially-induced TBI delivers inconsistent results. A significant variation was reported in the reduced movement speed of *C*. *elegans* after exposure to low-rate (5 Hz) ultrasound “shock waves” of uncontrolled acoustic spectral composition^52^. While the percentage of worms paralyzed and the slowing of those worms still moving was correlated with the ultrasound dose in that study, the uncontrolled propagation, reflection, and destructive interference of the ultrasound is likely responsible for the inconsistent results they observed. The uncontrolled reflections and interference likely caused even adjacent worms to experience completely different levels of “blast” exposure from the pulsed source. Others^54^ report trial devices using surface acoustic waves but with very few worms and a likewise uncontrolled exposure environment.

In any case, to obtain consistent exposure in the worm model, a proper worm-on-a-chip device that confines the worms into a microfluidic system should be designed to ensure homogeneous exposure of the individual worms to the ultrasonic pulse. Microfluidics has been a useful tool for housing and studying *C*. *elegans* for nearly a decade^55,56^, even for the analysis of neuronal cell behavior^57^. *C*. *elegans* can be introduced into the microfluidic device in large numbers, sorted and individually isolated into traps, and grown and tested as desired in a controlled environment within a structure amenable to microscopy. Furthermore, the design of the microfluidic device can be arranged such that the memory of the worm and its physical motility may be assessed entirely within the device^55^.

Surface acoustic wave (SAW) devices can be readily integrated with most of the current worm-on-a-chip microfluidic systems, potentially leading to a novel high-throughput method for studying bi-mTBI in individual *C*. *elegans* and screening potential drugs and therapies in the treatment of TBI in humans. However, more information is needed on what the effects are from SAW on the worms and whether these effects can be identified as significant upon their learning. SAW generated at frequencies ranging from MHz to GHz has been used for a broad array of phenomena in microfluidics including actuation and manipulation of fluids, particles, cells, and organisms^58–61^. However, the effect of high-acceleration SAW irradiation on the behaviour of *C*. *elegans* has just begun to be studied.

Unlike conventional ultrasonic devices, most of the acoustic energy associated with the SAW is confined to within 3–4 wavelengths of the substrate surface, and the piezoelectric material of choice is the non-hysteretic single-crystal lithium niobate, which has relatively poor piezoelectric coupling compared to lead zirconate titanate, but offsets this disadvantage with a significantly lower acoustic attenuation^58,61,62^. This makes SAW extremely efficient in transferring energy from the piezoelectric crystal to the fluid. In addition, the low power required (~1 W), at least an order of magnitude less than with conventional ultrasonic resonators, facilitate fabrication of miniaturized, low power portable devices consistent with high-throughput, lab-on-a-chip techniques. SAW devices operate at 10–100 MHz, much higher than conventional bulk ultrasonic resonators (10 kHz–1 MHz) and consequently cavitation is claimed to be prevented. Using continuous ultrasound, the threshold frequency for cavitation induced by any nucleation site of 10 nm or larger in water is 4.6 MHz, according to our simple calculations based on the classic work of Neppiras and Noltingk^63^, and so while this appears to be true, it should be confirmed.

Therefore, in this study, and beyond past reports showing paralysis and morphological changes, we show how exposure to high frequency acoustic waves generated by our SAW apparatus can affect *C*. *elegans* in two ways analogous to bi-mTBI observed in humans and mammals: reduction of both mobility and short-term memory. We demonstrate the absence of cavitation as a means to induce these effects, and show the importance of acoustic streaming in avoiding the heterogeneous results reported so far in the literature. These results will be shown to support the idea of using SAW with *C*. *elegans* as a simple, early-stage model for TBI.

## Results

### Dose-dependent mobility of *C*. *elegans* in response to SAW

**Figure 1:**
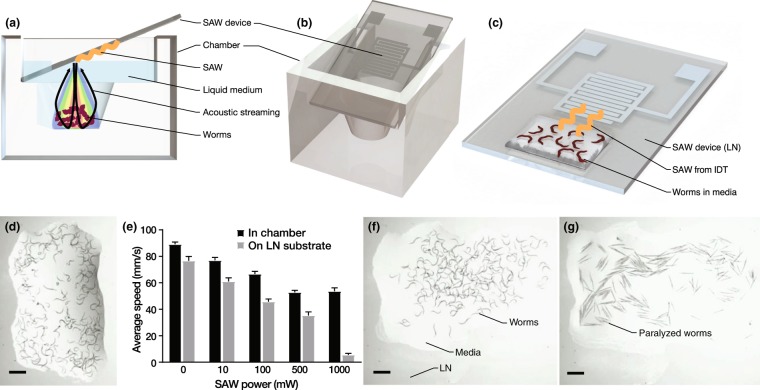
Two versions of the 20-MHz SAW *C*. *elegans* worm exposure concept were devised, one (**a**,**b**) introducing SAW-driven acoustic waves vertically into a chamber containing the *C*. *elegans*, and another (**c**) with the worms in a wetting sessile droplet of media placed directly upon the LN SAW device. The SAW device was placed at the Rayleigh angle (22°), as shown from the (**a**) side and (**b**) a perspective view, to vertically deliver the ultrasound in the well-based approach for 10 s. For scale, the (**a**–**c**) SAW device is 15 mm wide by 30 mm long. *C*. *elegans* worms are shown in a sessile droplet (**d**) prior to SAW exposure. The average speed of the *C*. *elegans* was then measured with them upon a solid agar plate as described in the methods. Upon increasing the SAW power in the 10-s trials to 500 mW, the worms exhibit a consistent and significant reduction in their movement speed. Here *N* = 3 independent trials were conducted with *n* = 300–500 worms per trial, and the error bars represent standard deviation. Normality of the data was assessed using the Shapiro-Wilk test with *p > *α* = 0.05 indicating the experimental data was normally distributed. The worms’ motility was always lower after SAW exposure in the (**c**) sessile droplet. As the SAW power (**c**) in the sessile droplet exceeds 500 mW for 10 s, the worms’ subsequent motility on an agar plate continues to decrease. Their motility remains nearly constant, however, after exposure to SAW (**a**,**b**) in the chamber. Direct (**e**–**g**) observation of the *C*. *elegans* in the (**c**) sessile droplet configuration showed the (**e**) unexposed worms to be (**f**) otherwise normal after experiencing 500 mW SAW for 10 s. By contrast, the worms (**g**) were completely paralyzed and became straight with only 5 s exposure to 1000 mW SAW. (**e**–**g**) Scale bars are 1 mm.

Utilizing our exposure apparatus illustrated in Fig. 1(a–d) and plating the *C*. *elegans* onto agar post-exposure, the worms’ average movement speed on agar decreased as the 20-MHz SAW input power and the associated amount of acoustic energy passed into the medium were increased as shown in Fig. 1. The dose-dependent mobility of *C*. *elegans* was studied for several SAW power levels and different exposure durations, from 5 s to 10 min, and an exposure time of 10 s was chosen as a balance between reducing any risk of heating effects and sufficient time for effects to arise from the SAW on the mobility of the worms.

Neither the worms’ movement nor behavior were measurably affected by SAW at applied powers below 50 mW. For moderate power (~100 mW), a reduction in the worms’ average motility was observed and acoustic streaming was insufficient to cause recirculation of the worms in the well (*see* Fig. 1(a,b)). Further increasing the SAW power to ~500 mW significantly reduced the worms’ average movement speed.

At this power, the worms are more effectively and uniformly exposed to acoustic radiation while freely suspended in the solution. This is a consequence of the formation of weak acoustic streaming. The acoustic streaming both translated and rotated individual *C*. *elegans* throughout the well. However, the observed reduction in movement speed with an increase in SAW input power did not extend to a higher input power of ~1000 mW. Beyond ~1000 mW, the fluid was found to begin atomizing and so we restricted our input power to 1000 mW or less. At 1000 mW, the worms were seen to be propelled by strong acoustic streaming from the exposure region in the well into regions of relative quiescence, and many became trapped in fluid stagnation points at the corners of the chamber. This appears to significantly *reduce* the net acoustic energy exposure for a typical worm despite the much higher input power.

To test this assertion, we instead exposed the worms to 10 s of SAW in a thin film (~1 mm thick) of buffer solution placed directly on the LN surface and entirely within the aperture of the propagating SAW (*see* Fig. 1(c,d)) before plating them onto an agar surface to measure their mobility. This both maximized the acoustic energy exposure and eliminated the possibility of worms escaping such exposure due to acoustic streaming. By using a thin fluid film of height less than a few wavelengths, there is insufficient distance over which the acoustic wave can be attenuated via viscosity in order to generate significant acoustic streaming^64^. Notably, the approximately linear reduction in the worms’ movement speed with increasing SAW power (Fig. 1(e)) is apparent all the way to 1000 mW in the thin film configuration, while the trend is weaker in the chamber at any power, and fails above 500 mW. Most importantly, otherwise motile and normal naïve worms as shown in Fig. 1(e) appeared essentially the same after 10 s of exposure to ~500 mW SAW (*see* Fig. 1(f)), with reduced motility as the only evidence of SAW exposure.

By contrast, the worms were completely paralyzed and straight from only 5 s of exposure to 1000 mW of SAW (*see* Fig. 1(g)) in the sessile droplet configuration (Fig. 1(c)). No paralysis of this form was ever observed from 10 s of exposure in the chamber (Fig. 1(a,b)) at any input power.

We seek to avoid paralysis while obtaining a consistent reduction in worm motility, believed to represent the sub-critical, principally behavioral injury in worms similar to bi-mTBI observed in humans and mammals: loss of function (mobility) without other obvious impairment. Consequently, 500 mW was chosen as the optimum SAW power while using the exposure chamber. This choice struck a balance between maximizing direct irradiation and minimizing acoustic streaming to achieve the maximum reduction in the worm’s speed of movement, a key loss of function, while simultaneously aiming to minimize the number of paralyzed worms. While the thin fluid film results are interesting in their own way, the problems of evaporation and difficulty in fluid handling prevented this configuration from being considered for the remainder of this study. The recovery of the worms’ mobility will be explored in the following section.

### Recovery of *C*. *elegans*’ mobility after SAW exposure

**Figure 2:**
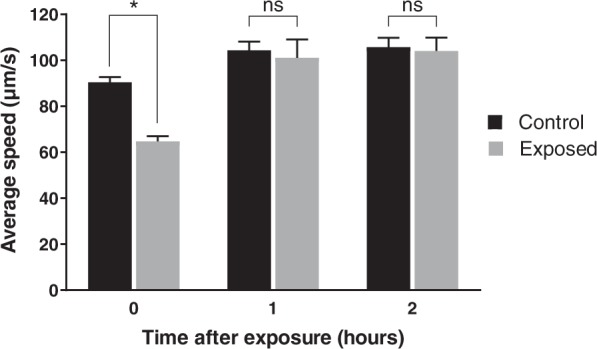
*C*. *elegans* motility recovers after 10 s of 20-MHz, 500 mW SAW exposure to become statistically indistinguishable with unexposed (control) worms in about one hour. Crowding (*see* text) was partially responsible for the initial reduction in motility for the control and 500-mW SAW-exposed worms, but a significant reduction in motility was observed due to SAW exposure alone. Mean (S.E.) values were obtained from *N* = 4 independent experiments, with *n* = 150–200 worms tested in each experiment (*p* < 0.05). Significance was based upon a two-way ANOVA over the *n* individual worm data and all *N* independent experiments for each reported time with $\alpha =0.05$, where ns implies *p* > 0.05, *p < 0.05, **p < 0.01, and ***p < 0.0005. Normality of the data was assessed using the Shapiro-Wilk test with *p > *α* indicating the experimental data was normally distributed.

As illustrated in Fig. 2, the worms’ mobility fully recovered within about 1 hour after 10 s of 20-MHz, 500 mW SAW exposure and deposition upon an agar plate, showing an average movement speed statistically indistinguishable (ns) to unexposed (control) worms. The average speed reported immediately after deposition upon the agar plate at 0 hours is much lower than after 1 or 2 hours, but this is in significant part due to the initial crowding of the worms. The worms were confluent in the M9 buffer before placement upon the agar, and so were constrained in their initial motion by crowding after placement; the control also exhibited a reduction in motility as a consequence. However, despite this effect, a significant reduction in worm motility was observed immediately after exposure (0 hours) that is directly attributable to SAW exposure.

It is important to ensure the exposed worms recover their mobility *before* being used for associative learning and memory assays that require worms to move along a certain distance and reach designated spots, so that the worms’ behavior is assessed in a manner analogous to the clinical assessment of bi-mTBI in humans, such as short-term memory loss and learning^65^. If the worms are mobile but still cannot move to designated spots, it is not because of the loss of mobility but instead is due to damage to their memory and ability to learn, the same as those observed in humans and mammals exposed to shock waves. Just as it would be unreasonable to judge a human’s brain impairment in a walking course with a broken leg, it is unreasonable to use reduced mobility worms for assays that conflate mobility with learning and memory.

### Effect of SAW on *C*. *elegans*’ learning and short-term memory: a proof-of-concept

**Figure 3:**
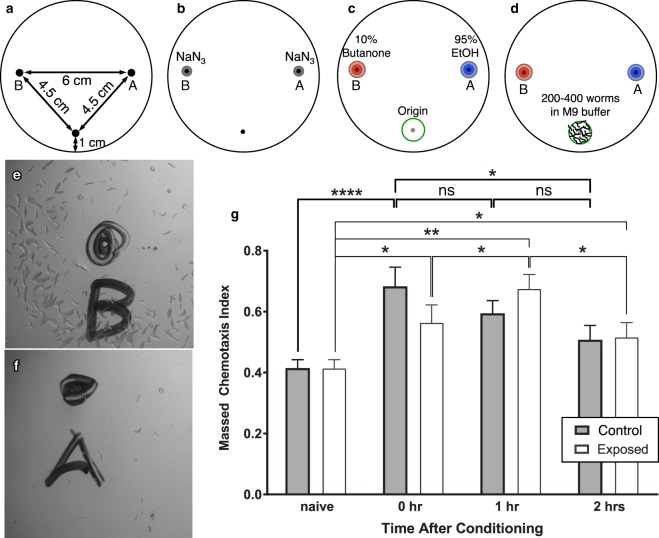
Chemotaxis assay (**a**–**f**) procedure and (**g**) results. The origin (at bottom), butanone (at left), and EtOH (at right) spots were (**a**) marked underneath a transparent plate. (**b**) First, 1 *μ*L NaN_3_ was added to each of the butanone and EtOH spots. (**c**) Then 1 *μ*L each of 10% butanone and 95% EtOH were respectively added to the spots on the left and right sides of the plate. (**d**) Immediately after being trained via the learning assay, 200 to 400 worms suspended in M9 buffer were then added to the plate over the “origin”. Upon reaching the (**e**) butanone and (**f**) EtOH spots, the worms were paralyzed and counted. In this way, the effect of 10 s of 500 mW 20-MHz SAW on associative learning and short-term associative memory of *C*. *elegans* worms may be (**g**) compared to worms unexposed to SAW. *Unexposed* worms showed significant learning ability (****p < 0.0002), and their short-term memory lasts for ~2 hours with an insignificant decline in their learning ability over that time (ns, p > 0.05 for 0–1 hr and 1–2 hrs). SAW-exposed worms exhibited a weakly significant change in the massed chemotaxis index (*p < 0.05) post-conditioning. The *SAW-exposed* worms showed a continued *increase* in learning ability to 1 hour after conditioning (*p < 0.05), followed by a *decrease* in learning ability from 1 to 2 hours after conditioning (*p < 0.05). The massed chemotaxis index significantly *increased* with respect to the unexposed (naïve) worms one hour after training (**p < 0.007). The naïve–0 hr, naïve–1 hr, and naïve–2 hr runs were each repeated four times (*N* = 4) for both the control and SAW-exposed conditions, with *n* = 200–400 worms for each and every repeat. Significance was based upon the entire data set with a two-way ANOVA with $\alpha =0.05$, where *p < 0.05, **p < 0.007, ***p < 0.0005 and ****p < 0.0002. Normality of the data was assessed using the Shapiro-Wilk test with *p > *α* indicating the experimental data was normally distributed.

As illustrated in Fig. 3, *C*. *elegans* worms *unexposed* to SAW showed a short-term memory profile consistent with past observations^65^. That is, after conditioning, wild-type worms’ chemotaxis to butanone increased significantly (****p < 0.0002) immediately after conditioning in comparison to their naïve state. This response to conditioning—typically associated with their memory of the training—was gradually lost over the next two hours, with an insignificant change (ns, *p* > 0.05) from both zero to one hour post-conditioning and one to two hours post-conditioning.

However, worms exposed to SAW showed different learning and short-term memory patterns. After conditioning, they exhibited only weakly significant (*p < 0.05) learning, and this learning actually *increased* in the hour after conditioning (*p < 0.05) and decreased from one to two hours post-conditioning (*p < 0.05). Further, the massed chemotaxis index correlated with their short-term memory differed between the exposed and unexposed worms: the SAW-exposed worms expressed the highest massed chemotaxis index fully one hour after training, in an apparently delayed but significant (***p < 0.007) learning response from conditioning.

### Absence of cavitation in SAW-induced reduction of *C*. *elegans*’ mobility

Cavitation—the formation and collapse of vapor bubbles from induced high positive pressure followed by negative pressure— and its resulting mechanical impact on *C*. *elegans* has been used to damage the neuronal structure and decrease *C*. *elegans*’ mobility^52,53^. Surprisingly, with SAW no such effect was seen. Cavitation, if present, was found to have no significant effect on the mobility of worms exposed to SAW and its associated high acceleration (10–100 Mm/s^2^) sound waves generated in the fluid surrounding the worms. While our calculations suggest the threshold frequency for cavitation induced by any nucleation site of 10 nm or larger in water is 4.6 MHz, based on the classic work of Neppiras and Noltingk^63^, the importance of the matter requires confirmation that cavitation is absent, or, if present, is not contributing to the observations.

**Figure 4:**
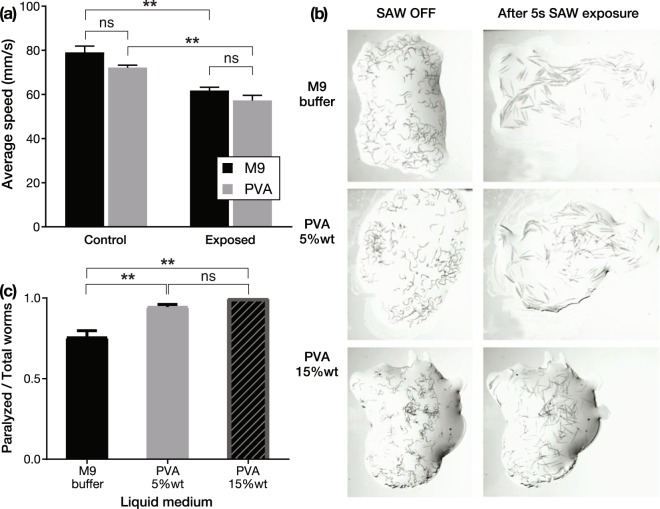
To determine if cavitation is a factor in the observed reduction in *C*. *elegans*’ motility, we exposed the worms to 10 s of 500 mW 20-MHz SAW in $10\,\mu \ell$ droplets of either polyvinyl alcohol or M9 solutions in the chamber configuration (*see* Fig. 1(a,b)). Polyvinyl alcohol, as prepared, is far more viscous than the M9 buffer solution with a consequent reduction in any ability to induce cavitation. The (**a**) effects of the SAW on the worms’ average speed in the two solutions were statistically identical (ns, *p* > 0.5), whether exposed to SAW or not. For (**a**), significance was based upon a two-way ANOVA upon data from $N=12$ independent experiments for the control and for the SAW-exposed worms, with *n* = 150–200 worms tested in each experiment, defining **p < 0.002. By increasing the SAW power to 1500 mW and switching to the (**b**) free fluid film configuration (*see* Fig. 1(c)), many of the worms were found to be paralyzed after only 5 s exposure, whether in M9, PVA 5% wt, or PVA 15% wt solutions. The (**c**) ratio of paralyzed to total number of worms in each experiment shows that switching from the less viscous M9 to the more viscous PVA produces a significantly (**p < 0.01) *greater* amount of paralysis, with insignificant (ns, *p* > 0.5) differences in paralysis between 5% wt and 15% wt solutions of PVA. Significance was based upon a two-way ANOVA upon $N=6$ independent experiments for each of the three liquid media choices, with *n* = 100–150 worms tested in each experiment, where ns implies *p* > 0.05, *p < 0.05, and **p < 0.01. In both (**a**,**c**) experiments, the normality of the data was assessed using the Shapiro-Wilk test with *p > *α* = 0.05 indicating the data were normally distributed.

The propensity to induce cavitation (if any) by SAW was assessed by simply replacing the M9 buffer with polyvinyl alcohol (PVA), which has much greater viscosity. Consequently, this change greatly reduces the induction, appearance, and intensity of cavitation^66,67^, while maintaining nearly the same acoustic impedance as water^68^. The speed of the worms remained similar despite the change in fluid, whether or not they were exposed to 500 mW SAW in the chamber (Fig. 4(a)).

By switching from the chamber configuration to the thin-film fluid configuration, and increasing the SAW power from 500 to 1000 mW, many of the worms were paralyzed. Figure 4(b,c) shows the worms’ paralysis regardless of whether M9, PVA 5% wt, or PVA 15% wt solutions were used. Interestingly, the worms’ paralysis *increased* with increasing viscosity, significantly so (**p < 0.01) in the switch from M9 to PVA. By contrast, switching between 5% wt and 15% wt solutions of PVA produced insignificant (ns, *p* > 0.5) differences in paralysis. If cavitation were a factor in the paralysis, the greater propensity of cavitation in the M9 would be expected to produce a greater number of paralyzed worms. The opposite occurs.

One aspect that must be checked is the relative acoustic impedance of these fluids. Potentially, the acoustic impedance $Z={\rho }_{f}{c}_{f}$, a product of the density ${\rho }_{f}$ and the speed of sound *c*_*f*_, could be so different between them that the effective transmission of acoustic energy into the fluid from the SAW could likewise be different, producing unexpected results. For example, perhaps the observed results are due to the PVA having a much lower acoustic impedance, with far less energy passing into the fluid from the SAW in the substrate.

To determine the acoustic impedance, we require the density and speed of sound. The density of each of the fluids is straightforward to measure, while the speed of sound may be determined from the Rayleigh angle ${\theta }_{{\rm{R}}}={\sin }^{-1}\,{c}_{f}/{c}_{{\rm{SAW}}}$ where *c*_*f*_ and *c*_SAW_ are the phase velocities of the sound in the fluid and of the SAW in the substrate, respectively: ${c}_{f}={c}_{{\rm{SAW}}}\,\sin \,{\theta }_{{\rm{R}}}$. The Rayleigh angle can be determined by measuring the angle of acoustic streaming produced within the fluid placed upon the SAW-generating substrate, visualized by dye tracing the flow from the acoustic source. In our measurements, deionized water (DIW), M9 buffer and PVA 5% weight produce Rayleigh angles of 22.0° ± 0.5°, 24.0° ± 0.5°, and 27.0° ± 0.5°. Since the SAW velocity is 3990 m/s in the LN substrate, this produces a sound speed in the fluid of *c*_*f*_ = 1490, 1620, and 1810 m/s, respectively, all ±30 m/s. The PVA solution therefore has a higher acoustic impedance than DIW and M9 as a result of its greater density and sound speed. This actually improves the transmission of sound into the fluid from the SAW in the substrate, so the change in the acoustic impedance is not responsible for the observations.

## Discussion

If acoustic streaming could be considered to be a contributing factor to the worms’ paralysis, it is insignificant (Fig. 4). Acoustic streaming is much weaker as the viscosity is increased from M9 to the PVA solutions, particularly the 15% wt solution, yet the paralysis effects are stronger with increasing viscosity. Furthermore, changes in the acoustic impedance were shown to not contribute to the observed effects. Finally, we find that cavitation is not responsible for the observed effects.

Therefore, it can be concluded that the sole remaining contributor from the SAW on the worms, large acceleration from the acoustic radiation propagating through the fluid, is the main injurious component. This effect appears to be responsible for the observed paralysis at high SAW intensity and the observed learning delays at lower SAW intensity induced in our worm-on-a-chip device. However, it is important to remember that acoustic streaming can aid in improving the homogeneity of the *C*. *elegans*’ exposure to the ultrasound by transporting them across the testing chamber, and can therefore be beneficial in improving the consistency of the results one may obtain.

Exposure to SAW-driven high frequency ultrasound affects the worms’ ability to move, with paralysis and morphological changes consistent with results shown in the literature^54^. Though past studies have indicated ultrasound appears to have some effect on *C*. *elegans* behavior and morphology^52–54^, in those studies where more than a few worms have been utilized, the results have lacked statistical power likely due to the heterogeneity of the ultrasound that the worms have been exposed to.

We furthermore show that exposure to SAW-induced ultrasound significantly affects associative learning and short-term memory, and that neither cavitation nor acoustic streaming is responsible for the observed effects, producing a potentially useful model for bi-mTBI in demonstrating a *delayed* learning ability after conditioning due to SAW exposure.

Regardless, the mechanism inducing these effects remains unknown. Further work is needed—and several studies are underway^69^—to determine the mechanisms underpinning ultrasonic neuromodulation before it may be considered a useful tool in neuroscience research. The current study indicates that SAW may prove to be useful in conjunction with *C*. *elegans* in studies that require the induction of significant effects in learning and memory.

## Methods

### Exposure apparatus

As depicted in Fig. 1, a single port, 25-finger pair interdigital transducer (IDT) 20 MHz SAW device with a 5 mm aperture was used in this work. The widths of the fingers and gaps between them were set at *λ*/4 = 50 *μ*m to define the SAW wavelength at *λ* = 200 *μ*m. Double-side polished 500 *μ*m thick, 127.68° *y*-rotated cut, *x*-axis propagating single-crystal lithium niobate (LN) was used for the substrate. Sputter deposition of 10 nm Cr/1 *μ*m Al was followed by ultraviolet photolithography with AZ 1518 photoresist (Microchem, Westborough, MA) and AZ 400 K developer (Microchem, Westborough, MA) and wet etching to fabricate the IDTs. The Rayleigh surface acoustic wave (Fig. 1(b,c)) was generated from a sinusoidal electric field generated by a signal generator (N9310A, Agilent Technologies, Santa Clara, CA) and applied via an amplifier (10W1000C, Amplifier Research, Bothell, WA). The input electrical signals were measured at the device using an oscilloscope (Wavejet 332/334, LeCroy, Chestnut Ridge, NY, USA), and the resulting Rayleigh wave was measured using a laser Doppler vibrometer (UHF-120, Polytec GmBH, Waldbronn, Germany).

The exposure chamber shown in Fig. 1(a,b) was molded from polydimethylsiloxane cast in a 3D-printed mold, and designed to hold the SAW device at an angle to orient the sound radiated from it into the media containing the worms. The sound was made to propagate downwards and perpendicular to the chamber’s bottom surface, where the worms were placed, accomplished by mounting the SAW device at the Rayleigh angle ${\theta }_{R}={\sin }^{-1}({c}_{l}/{c}_{s})\approx 22^\circ$. When the SAW comes into contact with the liquid underneath it, the acoustic energy diffracts into the drop due to the mismatch between the sound velocity in the substrate, *c*_*s*_ = 3990 m/s and the liquid, *c*_*l*_ = 1485 m/s for water.

To test the hypothesis that cavitation is not responsible for the effects observed from the SAW exposure on the worms’ behavior, as shown in Fig. 4, three different media were chosen with significantly different viscosities and densities. The standard M9 buffer, less viscous polyvinyl alcohol (PVA) (5% wt), and highly viscous PVA (15% wt), and then exposed for 5 s at 1.5 W SAW power. This enables the testing of the hypothesis whether the cavitation is an injurious component of the SAW-driven ultrasound.

#### *C*. *elegans* model

*C*. *elegans* were cultured using standard conditions^46^, with the details of their culturing and use as follows. All chemicals and consumables were obtained from Fisher Scientific (Carlsbad, CA USA) unless otherwise noted. The N2 worm strain was used for all studies and purchased from the Caenorhabditis Genetics Center (CGC; University of Minnesota, Minneapolis MN). Nematodes were grown and maintained on nematode growth medium (NGM) agar plates containing a lawn of the bacterium Escherichia coli (OP50). NGM agar (3 g NaCl, 17 g bactoagar (BD–DFO140010), and 2.5 g bactopeptone (BD-211677) in 2 L) was autoclaved, post autoclaving, the flask was cooled in a 55 °C water bath and 1 mL of 1 M CaCl_2_, 1 mL of 5 mg/ml cholesterol in ethanol, 1 mL of 1 M MgSO_4_, and 25 mL of 1 M KPO_4_ buffer were added to the cooled agar. The agar was then poured onto plates and the plates were dried at room temperature overnight prior to seeding with OP50. The OP50 mixture was cultured overnight. After measuring out 100 mL of sterile Miller’s LB Broth (Corning), one colony of OP50 (CGC) was added to the flask. The culture was left to grow overnight at 37 °C in an incubator with constant shaking. 50–250 *μ*L of the OP50 culture was added to the center of the dried plates. After seeding the plates with OP50, the plates were again dried overnight at room temperature prior to storage at 4 °C until use.

Age synchronization of worms was achieved via bleaching. Worms were allowed to grow until the adult stage. Gravid worms were recovered by washing plates with M9 buffer (3 g KH_2_PO_4_, 5.8 g Na_2_HPO_4_, 0.5 g NaCl, and 1 g 1 M NH_4_Cl to 2 L, autoclaved and then 2 mL of 1 M MgSO_4_ and 100 *μ*L of 1 M CaCl_2_ was added) into 15 mL conical tubes. These worms were pelleted via centrifugation (1500 rpm for 2 minutes) and the supernatant was discarded. The worms were washed 2–3 more times with M9, repeating the centrifugation and supernatant removal steps. After the last washing step, a bleaching solution was added to each tube (3.5 mL H_2_O, 0.5 mL 5 M NaOH, and 1 mL sodium hypochlorite). The tubes were then placed on a gentle rocker for 7 minutes. Immediately after the 7 minute agitation step, the tubes were filled with 10 mL of M9 to stop the reaction and prevent any harm to the eggs. Next, a quick centrifugation step was performed (~1500 rpm for 1 min) and the supernatant was aspirated. The pellet was washed three more times with M9 buffer, and after the final wash, the pellet was resuspended in 200 *μ*L of M9 and placed on seeded agar plates. The worms remained on the seeded agar plates for 48 hours at which time the worms were in the L4 stage and utilized for the experiments. The nematodes were kept in 20 °C incubator. All experiments, unless specifically noted, were run on nematodes age synchronized to L4.

### Locomotion data collection

About 200–300 worms dispersed in $100\,\mu \ell$ M9 solution^70^ were first placed at the bottom of the exposure chamber. An additional $400\,\mu \ell$ M9 was added to the chamber to bring the liquid surface into contact with the SAW device (Fig. 1(a,b)). After exposure, *C*. *elegans* were rapidly removed using a pipette and dispensed drop by drop on to a polyethersulfone filter membrane (47 mm diameter, 0.22 *μ*m pore size, Millipore, CA, USA) that was placed on the top of a vacuum filtration system comprised of a 150 ml filter bottle unit with 0.22 *μ*m Millipore top filter (Millipore Express Plus 0.22 *μ*m, Millipore, Temecula, CA). Once the liquid was removed, the membrane was removed and flipped over on a 6 cm agar plate seeded with OP50 *E*. *coli*. After a few seconds the membrane was removed, leaving most of the worms behind on the agar plate. The plate was immediately placed under a microscope (Leica KL200 LED, Leica Microsystems Inc., Buffalo Grove, IL, USA) and 1-min videos were then captured at 10 frames per second. After decompression and conversion to the AVI format, the videos were converted to 8-bit grayscale; background subtraction was performed using ImageJ and the resulting movie was thresholded to black-and-white using the automatic Otsu thresholding algorithm^71^. Next, the videos were analyzed using ImageJ (National Institutes of Health, Bethesda, Maryland) with the ImageJ plug-in wrMTrck^72,73^ to determine the average speed of movement of the *C*. *elegans*, reported in Fig. 1 as the average speed. The worms’ average movement speed was analyzed using a two-way ANOVA statistical method with $\alpha =0.05$, where *p < 0.05. Normality of the data was assessed using the Shapiro-Wilk test with *p > *α* indicating the experimental data was normally distributed.

### Associative memory assay

An associative learning and short-term memory assay similar to what was developed by A. Kauffman, *et al*.^65^ was used in this study. The memory assay involves three main steps: starving, short-term (1 hour) associative memory training, and a chemotaxis assay. The chemotaxis assay was performed on both naïve worms and non-naïve, trained worms immediately (0 hours), 1 hour, and 2 hours after training. More than 200 young adult wild-type worms were used per assay. Worms were first washed off of the high growth media (HGM) plates seeded with OP50 *E*. *coli* by pouring M9 buffer onto the plates. Then, the M9 buffer/worm mixture was transferred into a 15 mL conical tube where the worms were allowed to settle by gravity and the supernatant was removed by vacuum. Some of the worm population was used for the naïve chemotaxis assay while the remainder were kept in the conical tube in M9 buffer for 1 hour at room temperature to be starved. At the end of the starvation period, the supernatant M9 buffer was removed and worms were transferred to three training plates (>200 worms in each plate) seeded with OP50 *E*. *coli* and $2\,\mu \ell$ of 10% butanone (in 95% ethanol) streaked on the inside of the lids of the plates. After 1 hour of associative learning (food-butanone association), the lids were washed 2–3 times with M9 buffer/water to remove the butanone. Trained worms were washed off of one of the plates for the 0 hour time point chemotaxis assay, and the two other plates were kept at room temperature for 1 and 2 hours to await their later chemotaxis assays.

For the chemotaxis assay, as shown in Fig. 3, a $1\,\mu \ell$ paralyzing agent, 1 M NaN_3_, was first spotted at the control spots followed by $1\,\mu \ell$ each of 95% ethanol and 10% butanone on top of the previously spotted NaN_3_. Next, the worms were transferred to the “origin” spot and the excess M9 buffer was removed using the twisted corner of a KimWipe. The total number of the worms was counted by capturing an image of the worms at the origin immediately after the worms were released onto the assay plate. Then, the closed-lid chemotaxis assay plate was incubated for 1 hour at room temperature. Finally, the worms were counted by taking images of the origin, ethanol, and butanone spots, and the massed chemotaxis index, $CI=[({N}_{{butanone}})-({N}_{ethanol})]$/$[({N}_{total})-({N}_{origin})]$, was computed from this information^65^. The chemotaxis index was calculated for naïve worms and worms after 0, 1, and 2 hours of associative memory training. Identical associative learning and chemotaxis (short-term associative memory) assays were conducted for the control (wild-type, unexposed *C*. *elegans*) and SAW-exposed worms. In the latter case, the sole difference in the process was the initial exposure of the naïve worms to SAW before the associative memory training and chemotaxis assay.

The massed chemotaxis index data was analyzed using a two-way ANOVA statistical method with $\alpha =0.05$, where no significance (ns) refers to *p* > 0.05, *p < 0.05, **p < 0.01, and ***p < 0.0005. Specific changes in the definitions of the significance values are noted in the results where relevant. Normality of the data was assessed using the Shapiro-Wilk test with *p > *α* indicating the experimental data was normally distributed.
